# Substrate-specific transcription of the enigmatic GH61 family of the pathogenic white-rot fungus *Heterobasidion irregulare* during growth on lignocellulose

**DOI:** 10.1007/s00253-012-4206-x

**Published:** 2012-06-21

**Authors:** Igor Yakovlev, Gustav Vaaje-Kolstad, Ari M. Hietala, Emil Stefańczyk, Halvor Solheim, Carl Gunnar Fossdal

**Affiliations:** 1 Norwegian Forest and Landscape Institute, P.O. Box 115, 1431 Ås, Norway; 2 Department of Chemistry, Biotechnology and Food Science, Norwegian University of Life Sciences, 1432 Ås, Norway

**Keywords:** *Heterobasidion* spp., Glycoside hydrolases, GH61, Wood degradation, Gene expression

## Abstract

The GH61 represents the most enigmatic Glycoside Hydrolase family (GH) regarding enzymatic activity and importance in cellulose degradation. *Heterobasidion irregulare* is a necrotizing pathogen and white-rot fungus that causes enormous damages in conifer forests. The genome of *H. irregulare* allowed identification of ten *HiGH61* genes. qRT-PCR analysis separate the *HiGH61* members into two groups; one that show up regulation on lignocellulosic substrates (*HiGH61A*, *HiGH61B*, *HiGH61D*, *HiGH61G*, *HiGH61H*, and *HiGH61I*) and a second showing either down-regulation or constitutive expression (*HiGH61C*, *HiGH61E*, *HiGH61F*, and *HiGH61J*). *HiGH61H* showed up to 17,000-fold increase on spruce heartwood suggesting a pivotal role in cellulose decomposition during saprotrophic growth. Sequence analysis of these genes reveals that all *GH61*s except *HiGH61G* possess the conserved metal-binding motif essential for activity. The sequences also divide into groups having either an insert near the N terminus or an insert near the second catalytic histidine, which may represent extensions of the substrate-binding surface. Three of the *HiGH61*s encode cellulose-binding modules (CBM1). Interestingly, *HiGH61H* and *HiGH61I* having CBM1s are up-regulated on pure cellulose. There was a common substrate-specific induction patterns of the *HiGH61*s with several reference cellulolytic and hemicellulolytic GHs, this taken together with their low transcript levels on media lacking lignocellulose, reflect the concerted nature of cell wall polymer degradation.

**Electronic supplementary material:**

The online version of this article (doi:10.1007/s00253-012-4206-x) contains supplementary material, which is available to authorized users.

## Introduction

The *Heterobasidion annosum sensu lato* species complex encompasses pathogenic white-rot fungi primarily active in conifer forests that inflict massive economic and ecological losses due to tree mortality and wood decay (Woodward et al. 1998). The *Heterobasidion* complex comprised three Eurasian (*H. annosum* sensu stricto, *Heterobasidion parviporum*, and *Heterobasidion abietinum*) and two North American (*Heterobasidion occidentale* and *Heterobasidion irregulare*) species, each with a different but overlapping host range (Dalman et al. 2010; Niemelä and Korhonen 1998; Otrosina and Garbelotto 2010). Recently, the genome of *H. irregulare* (formerly *H. annosum* North American P-type) was sequenced and partially annotated (Olson et al. 2012). The annotated gene models reveal a wide repertoire of genes encoding lignocellulose degrading enzymes and these include several members of the family of polysaccharide monoxygenases that are currently classified as family 61 of the glycoside hydrolases (GH61). The GH61s have been predicted to be responsible for the initial step in cellulose breakdown by white-rot fungi, disrupting the cellulosic structure allowing subsequent attack by traditional cellulases (Quinlan et al. 2011; Vaaje-Kolstad et al. 2010).

A wide variety of GHs are utilized by wood decay fungi in the degradation of cellulose in plant cell walls. So far, enzymes classified in families GH3, 5, 6, 7, 8, 15, 27, and 48 in the carbohydrate active enzymes database have all been shown to degrade this fibrous and recalcitrant polysaccharide (Cantarel et al. 2009). Until recently, the consensus model for efficient enzymatic decomposition of cellulose was based on the synergistic combination of processive exo-acting cellobiohydrolases and non-processive endoglucanases (Merino and Cherry 2007; Teeri 1997). A disputed, but widely claimed hypothesis for cellulolytic fungi is that also (unspecific) cellulose cleavage by hydroxyl radicals plays an important role in depolymerizing this recalcitrant substrate (Eastwood et al. 2011; Goodell et al. 1997). Such reactions are by some authors suggested to be mediated by Fenton chemistry involving oxidative enzymes such as cellobiose dehydrogenases (CDHs) as electron donors (Mason et al. 2003; Zamocky et al. 2006). However, recent progress in the field has widened the view on the enzymatic factors involved in the deconstruction of cellulose and how these enzymes work in concert. In 2005, a protein (CBP21) of the carbohydrate module 33 family (CBM33) with no apparent enzymatic activity was shown to promote the enzymatic hydrolysis of chitin (Vaaje-Kolstad et al. 2005a; Vaaje-Kolstad et al. 2005b), revealing the existence of non-hydrolytic enhancing factors for recalcitrant polysaccharides as postulated by Reese and co-workers more than 50 years earlier (Reese et al. 1950). The existence of similar enhancing factors for cellulose was actualized upon the finding that fungal proteins classified as family 61 GHs were structurally very similar to CBM33s, sharing a highly conserved metal-binding motif on the substrate-binding surface (Karkehabadi et al. 2008). A verification of the notion that CBM33s and GH61s had a similar function was revealed by a study showing synergy between cellulases and GH61 proteins in the degradation of cellulose (Harris et al. 2010). Harris et al. (2010) was the first to demonstrate a functional role for the GH61 residues forming the metal-binding site by mutagenesis, corroborating the earlier mutagenesis results by Vaaje-Kolstad on CBM33s metal ion-binding histidines (Vaaje-Kolstad et al. 2005b). Recently, a key study on CBM33s acting on chitin showed that these proteins actually are metalloenzymes that cleave the polysaccharide chain through an oxidative mechanism that depends on an external electron donor (Vaaje-Kolstad et al. 2010). Importantly, the latter study also showed that the efficiency of substrate degradation could be greatly improved by providing an external electron donor. The generality of this enzymatic mechanism for cleavage of recalcitrant polysaccharides was recently shown for a CBM33 member (CelS2) that was proven active on cellulose (Forsberg et al. 2011). Shortly after publication of the studies on CBM33 enzymes, the oxidative reaction mechanism was also shown to apply for GH61 enzymes on cellulose (Phillips et al. 2011; Quinlan et al. 2011; Westereng et al. 2011). GH61s were in addition determined to specifically bind copper, thereby classifying this group of enzymes as copper-dependent monooxygenases (Phillips et al. 2011; Quinlan et al. 2011). Interestingly, it has also been shown that CDHs (naturally secreted in concert with GH61) can act as the external electron donor for GH61 enzymes (Langston et al. 2011; Phillips et al. 2011), indicating a more realistic role for these enzymes than what has formerly been indicated in the field (Fenton chemistry) (Mason et al. 2003; Zamocky et al. 2006). It should be noted that the GH61 proteins were originally classified based on measurement of very weak endo-1,4-β-d-glucanase activity in one family member (most likely endoglucanase activity). It is now obvious that both CBM33 and GH61 enzymes are erroneously classified and belong to the same group of polysaccharide oxidizing enzymes.

The main aim of the current study is to describe the relationship of GH61 members in *H. irregulare* and profile their transcriptional regulation and potential co-regulation with other hydrolytic cell wall degrading enzymes upon fungal growth in different natural and defined lignocellulose substrates mimicking the feeding and infectious stages. Transcript level profiles of the GH61s of *H. irregulare* were examined during growth on *Picea abies* (Norway spruce) heartwood and reaction zone (xylem defense tissue) in order to examine the predominant saprotrophic mode in this host and fungal response to the host defense reaction. Transcript levels during growth on *Pinus sylvestris* (Scots pine) sapwood were also examined, since sapwood is the tissue colonized by *H. irregulare* in this species. Finally, abundance of *GH61* transcripts was analyzed upon *H. irregulare* growth on pine heartwood, a host tissue heavily impregnated with antifungal compounds that allow only modest fungal growth. As controls, Hagem medium was used to examine for the *H. irregulare* gene expression when feeding on simple sugars and microcrystalline spruce cellulose was used to examine the gene expression on a cellulose substrate devoid of lignin.

## Materials and methods

### Samples, strain and culture conditions

The *H. irregulare* strain TC-32-1 subjected to genome sequencing by the DOE Joint Genome Institute (JGI) was used as the fungal strain in the inoculation experiment (Olson et al. 2012). The TC-32-1 strain is available from the culture collection Norwegian Forest and Landscape Institute, P.O. Box 115, N-1431 Ås, Norway. Liquid Hagem (0.5 g NH_4_NO_3_, 0.5 g KH_2_PO_4_, 0.5 g MgSO_4_ × 7H_2_O, 0.038 g (200 μM) MnSO_4_ × H_2_O, 0.8 mL Fe(II)Cl_2_ × 4H_2_O (1 % aqueous solution) and 5 g malt extract/1 L ddH_2_O) was prepared to be used as a basal medium for all treatments. The pH was adjusted to 4.5 with 1 M H_2_SO_4_, and after autoclaving, filter-sterilized thiamine HCl (0.1 mg/1 L) was added. To prepare the inoculum, the fungus was grown on 2 % malt extract agar for 3 weeks at 21 °C in darkness. Conidiospores were collected from the cultures into liquid Hagem and the spore concentration of the inoculum suspension was adjusted to 300,000 conidia/mL liquid Hagem with the aid of a light microscope and Bürker chamber. To prepare the experimental system, either 2 g of microcrystalline spruce cellulose powder (Sigma-Aldrich, #22182), 1 g of milled (IKA mill 10.2 impact grinding head, IKA Werke, Staufen, Germany) and gamma-sterilized heartwood or reaction zone tissue of Norway spruce (*P. abies*), or sapwood or heartwood of Scots pine (*P. sylvestris*) was weighed in a standard 9-cm-diameter Petri dish under aseptic conditions. The wood particles used were about 0.5 mm in size. For each Petri dish, substrates were wetted with 9 mL of the inoculum suspension, this small volume allowing aeration of the hygroscopic cellulose and natural lignocellulose (wood) cultures. As a reference treatment, Petri dishes containing only 9 mL of the inoculum suspension were also prepared. Three replicates were prepared for each substrate and the experiment was harvested after an incubation period of 21 days at 21 °C in darkness. The samples were stored in −80 °C until further processing.

### *GH61* gene annotation, peptide structure and phylogenetic analyses

Initial search for GH61 family genes was done within the JGI *Heterobasidion annosum* v2.0 genome web-portal (http://genome.jgi-psf.org/Hetan2/Hetan2.home.html) by means of search facilities using keywords: IPR005103 (InterPro Glycoside hydrolase, family 61 ID) and/or PF03443 (HMMPfam:Glyco_hydro_61 ID) as search terms. Selected gene models were confirmed using JGI *H. annosum* NCBI blast server with BLASTP search against the National Center for Biotechnology Information (NCBI) GenBank (http://www.ncbi.nlm.nih.gov/) and the Conserved Domain Database (Marchler-Bauer et al. 2011). Detected putative homologs were characterized based on conserved domains and *E* values in comparison with known proteins. All gene models containing Glyco_hydro_61 pfam03443 or cl04076 motif were chosen for further study.

Signal protein sequences were predicted with SignalP 3.0 (http://www.cbs.dtu.dk/services/SignalP; Dyrløv Bendtsen et al. 2004).

All selected GH61 gene models and translated protein sequences were retrieved from the JGI Fungal Genome Sequence databases. The amino acid sequences were aligned using ClustalW (http://align.genome.jp/) and TCoffee (http://tcoffee.vital-it.ch/cgi-bin/Tcoffee/tcoffee_cgi/index.cgi) (Notredame et al. 2000), and obtained multiple alignments were adjusted manually with GeneDoc (http://www.nrbsc.org/gfx/genedoc/index.html). Similarities/identities between selected pairs of DNA or protein sequences were calculated using MatGAT (Matrix Global Alignment Tool) (http://bitincka.com/ledion/matgat/).

Phylogenetic and molecular evolutionary analyses of translated gene sequences (full ORF) of the selected *H. irregulare GH61* models were conducted using the MEGA5 software (Tamura et al. 2011). The evolutionary history was inferred using the neighbor-joining method. The bootstrap consensus tree inferred from 500 replicates was considered to represent the evolutionary history of the taxa analyzed. Branches corresponding to partitions reproduced in less than 50 % bootstrap replicates werecollapsed. The phylogenetic tree created was linearized assuming equal evolutionary rates in all lineages. The evolutionary distances were computed using the Poisson correction method and are in the units of the number of amino acid substitutions per site as the distance measurement unit. All positions containing alignment gaps and missing data were eliminated only in pairwise sequence comparisons (Pairwise deletion option).

### RNA isolation

Prior to RNA extraction from the inoculation experiment, reaction zone and heartwood samples were aliquoted into 2-ml Eppendorf tubes, and ground twice for 1 min each time at maximum speed) using a Retsch 300 mill (Retsch GmbH, Haan, Germany) with liquid nitrogen chilled containers and a 100-mg steel bead. Other substrates were ground with a pestle in a liquid nitrogen–chilled mortar. Total RNA was extracted from 100 mg powder of the pulverized wood samples using the MasterPure™ RNA Purification Kit (Epicentre, Madison, WI, USA, # MCR85102) following manufacturer recommendation and stored at −80 °C until further use. RNA was quantified using a micro-volume spectrophotometer NanoDrop 2000 (Thermo Scientific, Wilmington, DE, USA).

### cDNA synthesis and qRT-PCR

Transcript levels of selected genes were determined for mycelium growing on six different media (Hagem, cellulose, spruce heartwood and reaction zone, and pine heartwood and sapwood) described above, using quantitative RT-PCR. Gene specific primers were designed using Primer3 (Rozen and Skaletsky 2000) and following criteria: melting temperature 70 °C and product size inferior to 120 bp. The list of studied gene homologs and their primer sequences are shown in Table S4 in the Electronic supplementary material (ESM). Total RNA was reverse transcribed (300 ng/reaction) using the TaqMan Reverse Transcription kit (Applied Biosystems, Carlsbad, CA, USA, # 8080234) in 50-μl-reaction volume. PCR amplification was performed in a 25-μl-reaction volume, using 2 μl of fourfold diluted cDNA solution as template, 12.5 μl of ×1 SYBR Green master mix and 200 nM of each primer. RT-PCRs were performed using the 7500 Fast Real-time PCR System (Applied Biosystems, Carlsbad CA, USA) with standard cycling parameters. All reactions were done in triplicate and no-template control was run for each primer pair. For data analysis, the arithmetic mean of two biological replicates was calculated. Target gene expression was normalized to geometric mean of genes encoding the *H. irregulare* actin (*HiAct*), α-tubulin (*HiαTub*) and ubiquitin-conjugating enzyme 2 (*HiUbc2*) (Table S4 and Fig. S1 in the ESM).

Absolute quantification was performed using 7500-system SDS software. The qRT-PCR Data were further processed in MS Excel and analyzed using the RT^2^ Profiler PCR Array Data Analysis Version 3.5 web portal from SABiosiences/Qiagen (Frederick, MD, USA) (http://pcrdataanalysis.sabiosciences.com/pcr/arrayanalysis.php) using portal defaults, to obtain p-values and correlation coefficients for each gene product (Tables S7 and S8 in the ESM).

### *GH61* gene amplification and sequencing

In order to confirm the predicted *GH61* gene models, the coding regions of the *GH61*s in *H. irregulare* and *H. parviporum* (a species primarily associated to Norway spruce) were PCR amplified and sequenced.

For *H. irregulare* RNA was obtained from colonized cellulose medium while for *H. parviporum* we used RNA from natural infection of Norway spruce (Yakovlev et al. 2008). Full-length doubled-stranded cDNAs were obtained from 1 μg of RNA using the SMART–PCR cDNA Synthesis Kit (Clontech, Palo Alto, CA, USA). PCR amplification of the full-length coding cDNA, from the start to the stop codons, was performed on a GeneAmp 9600 thermocycler (Perkin-Elmer Instruments, Shelton, CT, USA) using the Advantage 2 Polymerase Mix (Clontech). Primers were designed using the nucleotide sequences of selected *H. irregulare GH61* gene models (Table S5 in the ESM) and used for both species. The PCR products were analyzed in parallel on a 2.0 % agarose gel stained with ethidium bromide. Amplified products (single and multiple clear bands) were excised from gel, purified with the Qiagen MinElute Gel Extraction Kit (Qiagen, #28606), and sequenced directly from both sides using the corresponding cloning primers. The obtained sequences were assembled into contigs using the SecMan Pro module of DNAStar Lasergene sequence analysis software suite (DNASTAR Inc., Madison, WI). Contigs for each of the *Heterobasidion* species were aligned with *H. irregulare* gene model sequences and between species. Sequences of the *H. parviporum* cDNAs described in this study are available at the NCBI database under accession numbers JQ290102 to JQ290109.

### Molecular modelling

A sequence alignment representing the starting point of the modeling procedure was prepared by extracting the HiGH61A and TtGH61E (PDB ID: 3EII) (Harris et al. 2010) sequences from a ClustalW generated structure based multiple sequence alignment (MSA) generated for all HiGH61 sequences (see above) and converted to PIR format with “gap-only” columns removed. The alignment was used to build the 3D molecular model of HiGH61A, running MODELLER 9v4 (Eswar et al. 2007) and using the TtGH61E crystal-structure as structural template. The 3D models of HiGH61A were selected from 20 models, based on the value of the Modeller objective function, and further refined. The energy configuration outputs of the best-ranked models were used as starting parameters for loop optimization, followed by energy minimization using a constrained Cα backbone. The stereochemical quality and overall G-factor of the optimized model was calculated with Procheck (Laskowski et al. 1993) and the compatibilities of the models with overall folds were estimated with ProSA-web (Wiederstein and Sippl 2007). The root-mean-square deviation (RMSD) values in the Cα backbone positions between the HiGH61A model and 3EII was determined using the ‘super’ algorithm in PyMol (http://www.pymol.org) that was also used for generation of molecular graphics (DeLano 2002). Analysis of the accessible molecular surface was done using the WHATIF software (Vriend 1990).

## Results

### Sequence analysis and structural modeling of HiGH61 sequences of *H. irregulare*

In total, ten genes coding for candidate proteins of the glycoside hydrolase family 61 (GH61) were identified (Table S1 in the ESM). The *HiGH61* family genes are relatively dispersed throughout the *H. irregulare* genome and placed at scaffolds 01, 03, 05 (two genes), 07, 08, 09, 10 (two genes), and 13. The two genes, *HiGH61G* and *HiGH61I*, are adjacent to each other at scaffold 5 within <4 kb genomic interval with 46 % of identity (Table S2 in the ESM).

**Fig. 1:**
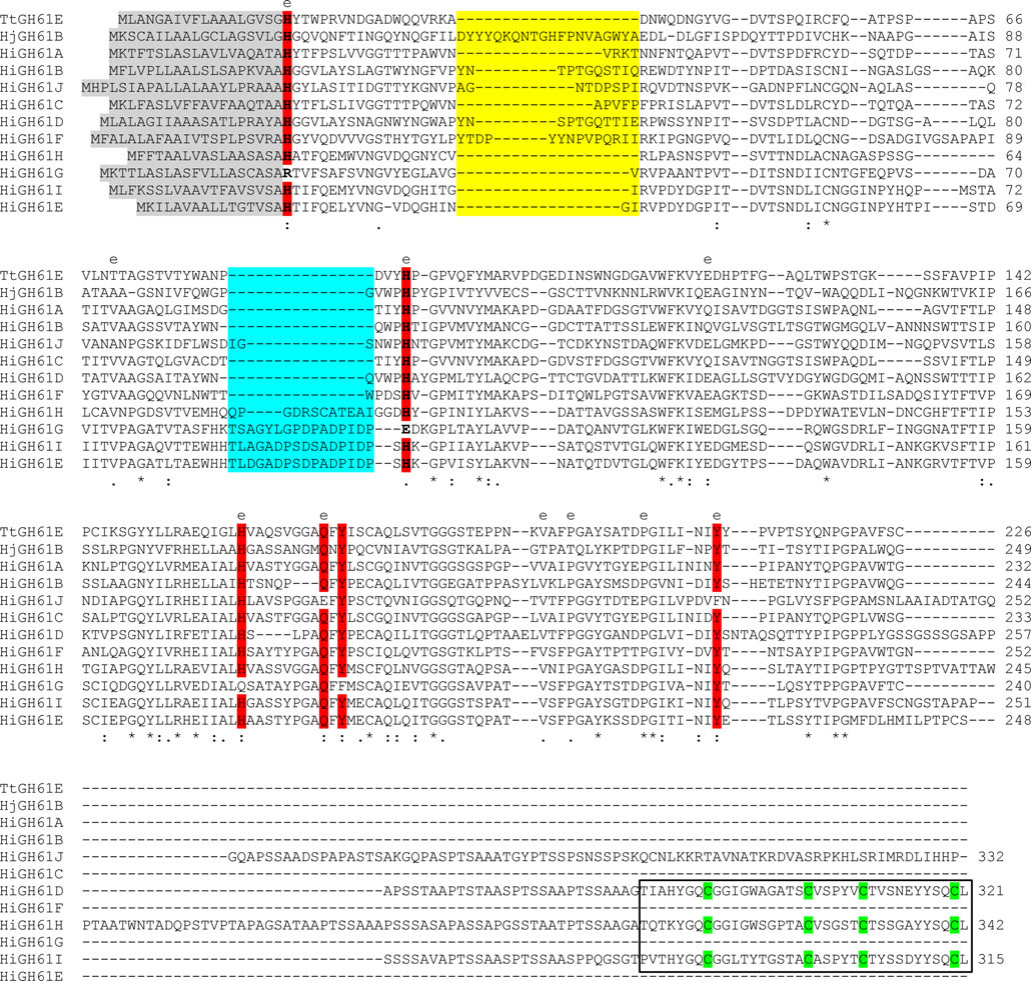
Multiple sequence alignment of HiGH61 sequences and sequences of GH61 proteins with known 3D structures (TtGH61E from *T. terrestris* and HjGH61B from *H. jecorina*). Regions with insertions/deletions are shaded *yellow* (region 1) and *blue* (region 2). Conserved residues clustered on the putative substrate-binding surface/active site are shaded *red*. Degree of conservation: *asterisks* fully conserved, *colons* highly conserved, and *full stops* moderately conserved. Conserved residues with solvent exposed side chains (inferred by analysis of the HiGH61A 3D model by WHATIF) are marked with an “*e*”. CBM1 domains are indicated by a *box* and the conserved cysteines within these modules are colored *green*

All the analyzed HiGH61 polypeptide sequences contain a 16–22 aa long secretory signal peptide at the N terminus. The deduced amino acids sequences of full-length *HiGH61* genes varied in length from 233 to 343 amino acids and the respective genomic sequences contained multiple exons (from 4 to 11) (Table S1 in the ESM). All mature HiGH61 polypeptide sequences share the conserved metal ion-binding site that contain two histidines included in the N terminus of the mature protein, except HiGH61G where these histidines have been replaced by arginine and glutamine, respectively (Fig. 1).

In addition to the conserved metal-binding motif, four proteins (HiGH61D, HiGH61H, HiGH61I, and HiGH61J) also possess an additional single domain at the C-terminal side of the GH6 module. For HiGH61D, HiGH61H, and HiGH61I, the extra domain was identified as a family 1 carbohydrate-binding module (CBM1; also known as fungal cellulose-binding domain) (Gilkes et al. 1991), whereas HiGH61J has a C-terminal extension of ∼35 residues of unknown function.

**Fig. 2:**
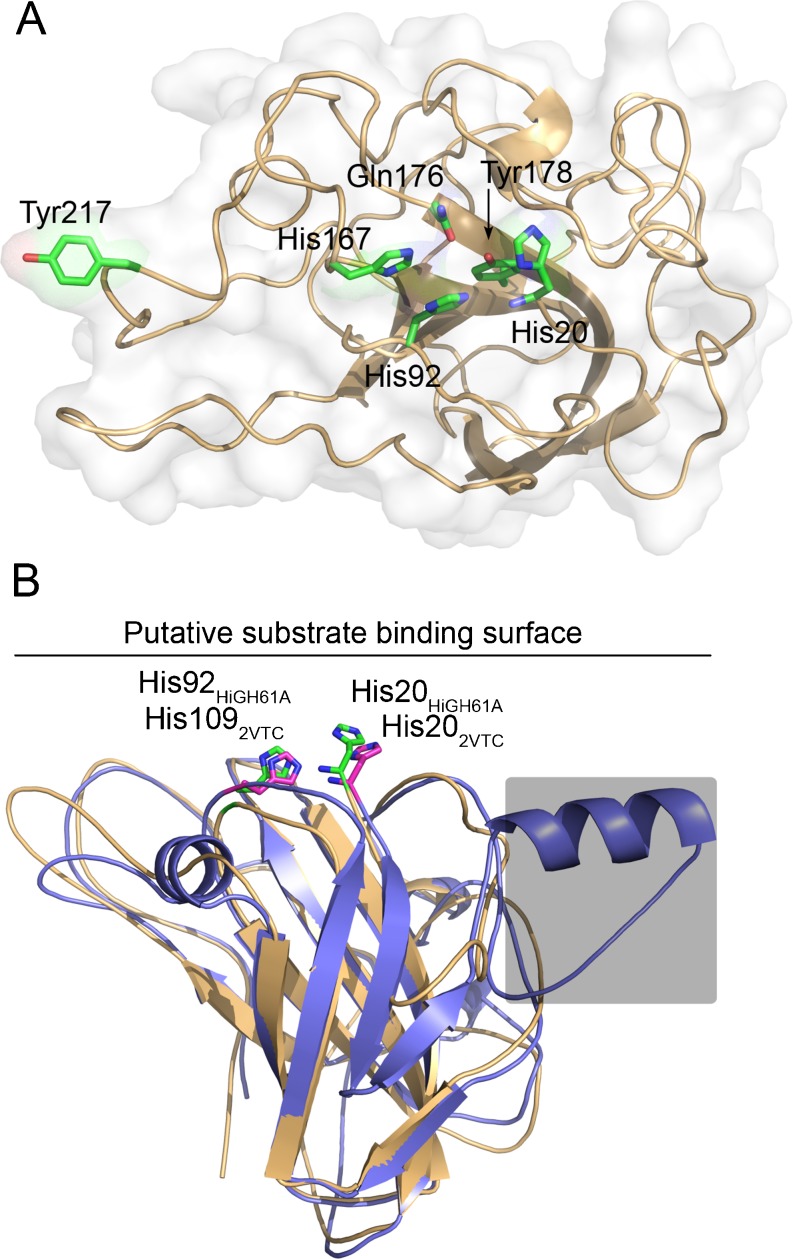
3D structural model of HiGH61A. **a** A superposition of the HiGH61A model (*light-orange cartoon*) and HjGH61B (PDB ID: 2VTC; *blue cartoon*) illustrating the binding surface extension seen in some of the HiGH61 proteins (sequence part covered by a *grey shaded box*; see also Fig. S1 in the ESM). The putative substrate-binding surface is seen from the side and also indicated by a *line*. The two conserved metal-binding histidines that are essential for GH61 activity (metal not shown) are shown in *stick representation* (colored *green* for HiGH61A and *magenta* for HjGH61B). **b** *H. irregulare*, a view looking down on the putative HiGH61A substrate-binding surface (i.e., rotated 90° around the *x*-axis compared with (**a**)) with the conserved, solvent-exposed amino acids shown in *green stick representation*. The protein is visualized in *light-orange cartoon representation* surrounded by a *transparent white molecular surface*

A MSA of the *H. irregulare* GH61 sequences was obtained by aligning the sequences to a profile consisting of a 3D-structural alignment of TtGH61E and HiGH61B from *Thielavia terrestris*; PDB ID: 3EII (Harris et al. 2010) and *Hypocrea jecorina*; PDB ID: 2VTC (Karkehabadi et al. 2008), respectively (Figs. 1 and 2). In order to aid and visualize the interpretation of the MSA, a 3D model of HiGH61A was made using the MODELLER software using the TtGH61E structure as template (41 % sequence identity and 3 % gaps). Analysis of the HiGH61A 3D model reveals that the majority of conserved residues of the HiGH61 isozymes are found internally in the protein where they most likely contribute to stabilizing the protein fold, similar to what is observed in a study on GH61s from *T. terrestris* (Harris et al. 2010). Analysis of the accessible molecular surface shows that only 11 of the 52 conserved residues have solvent exposed side chains, whereof six are present on the putative substrate-binding surface (Fig. 2). These include His20 and His92 that are involved in the metal-binding motif and additionally Gln176, His167, Ile215, and Tyr217 that are likely to play important roles in the catalytic mechanism/substrate binding of these proteins. Tyr217 is the only aromatic amino acid in the protein that is solvent exposed, much like what is seen for the chitin oxidizing enzyme CBP21 (Vaaje-Kolstad et al. 2005b), where it has been shown to be important for activity and substrate binding (Vaaje-Kolstad et al. 2005a).

Evaluations of stereochemical parameters of the final model showed no residues in the disallowed region of the Ramachandran plot (analyzed by Procheck), (Laskowski et al. 1993), overall G-factor obtained = −0.2, an RMSD value of the Cα positions between the HiGH61A model and TtGH61E of 0.18 Å (analyzed using the “super” algorithm in Pymol (DeLano 2002) and an overall good model quality (*Z* score = −5.12) when analyzed by the ProSA software designed for detection errors in protein structures (Wiederstein and Sippl 2007).

### GH61 phylogeny

**Fig. 3:**
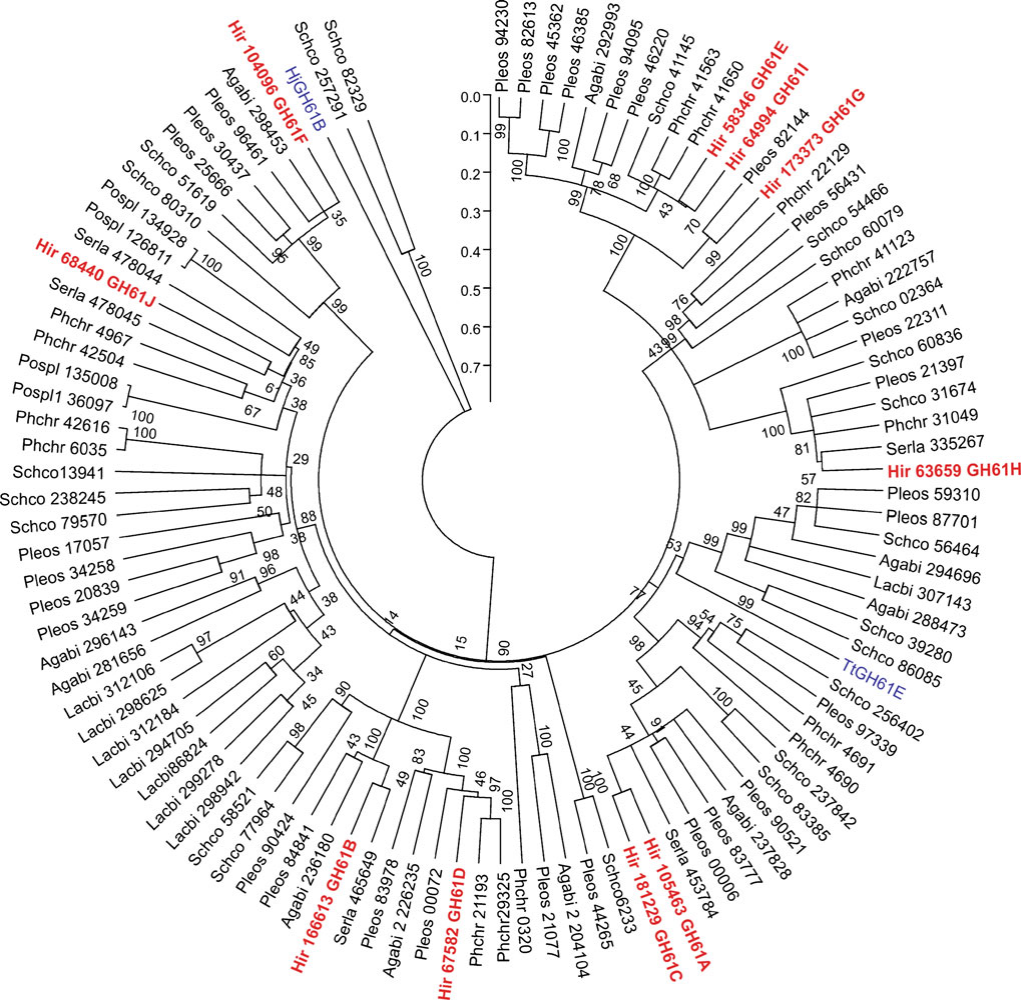
Phylogenetic tree of GH61 family proteins (full ORF amino acid sequences) available at the JGI fungal genomic database. Species abbreviation and protein ID are shown for each entry. The phylogenetic tree was constructed using MEGA5 as detailed in “Materials and methods.” The tree is drawn to scale, with branch lengths in the same units as those of the evolutionary distances used to infer the phylogenetic tree. Sequences from *H. irregulare* (Hir) are shown in red. Fungal species: *Agabi A. bisporus*, *Pleos P. ostreatus*, *Pospl P. placenta*, *Lacbi L. bicolor*, *Phchr P. chrysosporium*, *Serla S. lacrymans*, *Schco S. commune*, TtGH61E from *T. terrestris* and HjGH61B from *H. jecorina*

The phylogenetic tree was inferred from the alignment of most of the GH61 proteins found in the JGI genomic database using the neighbor-joining method (Fig. 3). In addition to the 10 GH61 protein sequences from *H. irregulare*, proteins from the saprotrophic white-rot fungi *Phanerochaete chrysosporium* (14), *Pleurotus ostreatus* (29), and *Schizophyllum commune* (14); the saprotrophic brown-rot fungi *Postia placenta* (4) and *Serpula lacrymans* (5); the mycorrhizal fungus *Laccaria bicolor* (14); and the leaf litter decayer *Agaricus bisporus* (11) were also included (Table S3 in the ESM). *H. irregulare* GH61 proteins clustered into five different diverse groups without obvious species or decay mode specificity. Two protein pairs stood out with very-high-sequence similarity; HiGH61E and HiGH61F with 61 % of identity and HiGH61A and HiGH61C having≈80 % of identity (Table S2 in the ESM). HiGH61A and HiGH61C grouped in the same branch as TtGH61E from *T. terrestris*.

### Comparison of GH61 family coding sequences between *H. irregulare* and *H. parviporum*

Amplification of *GH61* genes in *H. irregulare* samples produced in all cases single PCR products with expected product size, except for *HiGH61C* and *HiGH61E*, which showed faint PCR products. Amplification of *GH61* genes in *H. parviporum* samples produced clear single PCR products with expected product size only for *HpGH61G–HpGH61J*, multiple bands for *HpGH61A–HpGH61F* and no amplification product for *HpGH61C* and *HpGH61E* (Table S6 in the ESM). The obtained multiple bands for *H. parviporum*, when bright and clear on gel, were also sequenced. In all cases the sequences obtained from *H. irregulare* corresponded to predicted gene model sequences. The *GH61G–GH61J* gene sequences revealed clear single nucleotide polymorphisms between the two species, some being non-synonymous. The *HpGH61J* sequence showed a synonymous insert of 21 bp (7 aa). Most of sequenced fragments excised from *H. parviporum* samples with multiple PCR products were not related to *GH61* family genes or were not found in the *H. irregulare* genome.

### Transcription patterns of *H. irregulare* GH61 family genes

**Fig. 4:**
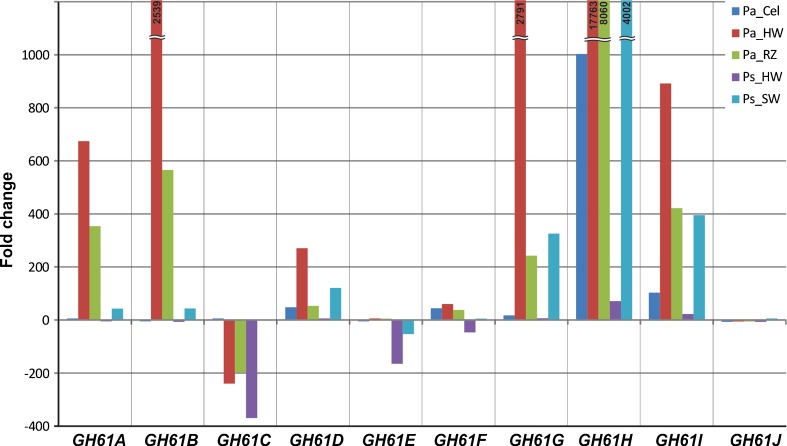
Transcript level profiles of GH61 polysaccharide oxidases of *H. irregulare* during fungal growth on different substrates from *P. abies* (*Pa*) and *P. sylvestris* (*Ps*). Substrates: *Cel* cellulose, *RZ* powdered reaction zone wood of Norway spruce, *HW* powdered heartwood wood of Norway spruce and Scots pine, *SW* powdered sapwood of Scots pine. Analysis was based on three biological replicates per culture medium. Transcript levels, normalized to the geometric mean of *HiAct*, *HiαTub*, and *HiUBC2* Ct values (endogenous controls), are shown as fold changes in relation to their normalized transcript levels on the control medium (liquid Hagem)

**Fig. 5:**
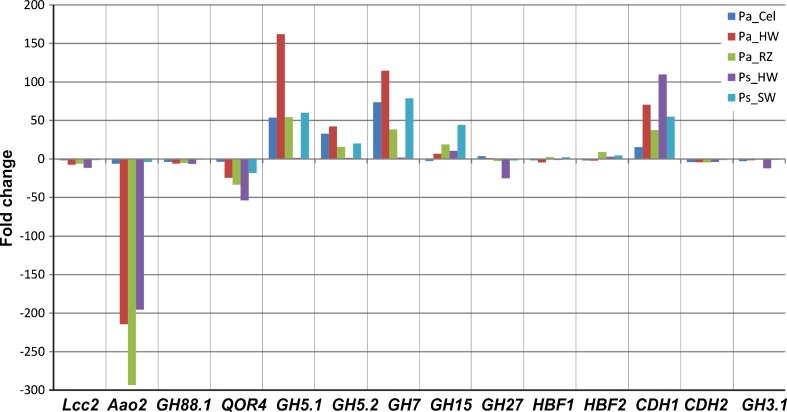
Transcript level profiles of selected lignocellulose active genes of *H. irregulare* (see legend of Fig. 4 for more information). *Lcc* laccase 2, *Aao* aryl-alcohol oxidase, *QOr* quinone oxidoreductase, *СDH* cellobiose dehydrogenase, *HBF* hydrophobin, *GH* glycoside hydrolase

Gene expression data of ten *HiGH61* family genes and twelve reference genes with carbohydrate active gene products were profiled on four natural substrates (liquid Hagem supplemented with heartwood and reaction zone of Norway spruce, sapwood and heartwood of Scots pine) and two defined substrates (cellulose supplemented liquid Hagem and sole liquid Hagem) using qRT-PCR (Figs. 4 and 5, respectively). The basal liquid Hagem medium was considered as control and all normalized gene expression data were related to transcript level on Hagem medium. All the *GH61* family genes were expressed but showed differential expression between the different substrates. Most of the *GH61* family genes, except *HiGH61C*, were up-regulated on spruce heartwood (up to ≈17,000-fold change (FC)) and reaction zone (up to ≈ 8 000 FC), the genes *HiGH61A, HiGH61B*, and *HiGH61G–HiGH61I* being highly induced on both substrates (above 200 FC). Similarly, most of the *GH61* family genes, excluding *HiGH61E*, were up-regulated on pine sapwood, the genes *HiGH61G–HiGH61I* showing the highest transcript levels (≥300–4,000 FC). On pine heartwood only *HiGH61H* showed the highest transcript levels (up to ≈80 FC) while the remaining *GH61* genes were either slightly induced or down-regulated (*HiGH61C*, *HiGH61D*, and *HiGH61E*). On cellulose, most of the *GH61* family genes, excluding *HiGH61H*, tended to increase.

In general, the highest transcript levels of *GH61* family genes were observed on spruce heartwood, followed by spruce reaction zone, pine sapwood, cellulose and pine heartwood. Among the profiled *GH61* genes *HiGH61H* clearly showed the highest induction, whereas *HiGH5.1* (a GH5 cellulase encoding gene) together with the *HiGH7* (GH7 cellulase) and *HiCDH1* (encoding a cellobiose dehydrogenase) showed the highest induction among the carbohydrate active reference genes.

*HiGH61A*, *HiGH61B*, and *HiGH61D–HiGH61I*; *HiGH5.1* and *HiGH5.2*; *HiGH7*; and *HiCDH1* appeared to be coordinately regulated on all substrates (Figs. 4 and 5; Table S7 in the ESM). *HiGH61C* showed similar tendencies in substrate-specific transcript level patterns with *HiGH27* and *HiLcc2*, whereas *HiGH61J* showed correlation in transcript level patterns with *HiGH15*, *HiGH88.1*, and *HiCDH2.*

### Transcription patterns of *H. irregulare* CDH family genes

Two cellobiose dehydrogenase genes have been described in *H. irregulare* genome. The proteins encoded by these genes are secreted and contain the common cellobiose dehydrogenase/carbohydrate-binding 9 domain (IPR015922) but differ significantly by sequence and structure. *HiCDH1* encodes a large protein of 775 aa containing N-terminal cellobiose dehydrogenase domain followed by a glucose-methanol-choline oxidoreductase domain (IPR007867) or FAD/NAD(P)-binding domain (SSF51905). *HiCDH2* encodes a smaller protein of 285 aa containing N-terminal cellobiose dehydrogenase domain followed by a carbohydrate-binding module 1 (CBM1) (PF00734).

Transcription patterns of the two *CDH* genes were different: while *HiCDH1* was upregulated on all substrates, *HiCDH2* was only slightly expressed and downregulated on all substrates. The maximum transcript levels for *HiCDH1* were observed on pine heartwood of spruce, followed by spruce heartwood, pine sapwood, and spruce reaction zone (Fig. 5). *HiCDH1* transcript levels correlated with transcript levels of most of *GH61* genes, except *HiGH61F* and *HiGH61J*. For *HiCDH2*-positive correlations in substrate-dependent transcript level, patterns were found with *HiGH61J* and two GH genes (*HiGH88.1* and *HiGH15*), but for all of these, the expression appears to be constitutive and largely unaffected by the treatments (Table S7 in the ESM).

## Discussion

The recent evidence for GH61/CBM33 proteins being oxidative polysaccharide cleaving enzymes (Forsberg et al. 2011; Phillips et al. 2011; Quinlan et al. 2011; Vaaje-Kolstad et al. 2010; Westereng et al. 2011) has established a new line of thought on how microbes depolymerize recalcitrant biomass. In this respect, *H. irregulare* is a typical representative of cellulolytic fungi as it possesses ten genes encoding GH61 enzymes (fungi with over 40 GH61 encoding genes have been reported, e.g., *Chaetomium globosum* (Longoni et al. 2012)). Sequence analysis of these genes reveals that all except HiGH61G possess the conserved metal-binding motif that is essential for activity (Fig. 1). The sequences also divide into groups having either an insert near the N- terminus (Fig. 1, yellow shading) or an insert near the second catalytic histidine (Fig. 1, blue shading), which both may represent extensions of the substrate-binding surface. Three of the enzymes also contain C-terminal cellulose-binding modules (CBM1), indicating direct targeting of crystalline cellulose.

The transcription data of the *HiGH61* genes divides this enzyme class into two different groups; one showing clear up regulation on some or all substrates (*HiGH61A*, *HiGH61B*, *HiGH61D*, *HiGH61G*, *HiGH61H*, and *HiGH61I*) and the other showing either down-regulation or unaltered gene expression compared with the control medium (*HiGH61C*, *HiGH61E*, *HiGH61F*, and *HiGH61J*). This grouping suggests that the fungus up- or down-regulate different sets of GH61s depending on the substrate used for growth, like in the various stages of necrotizing and saprotrophic growth on the host. Interestingly, all GH61s that have a CBD domain at the C-terminal (*GH61D*, *GH61H*, and *GH61I*) are up-regulated on all substrates except heartwood of Scots pine and are the only GH61s that show clear up regulation in the pure cellulose substrate (Fig. 4), indicating that at least these three GH61s are designed to specifically target cellulose surfaces. The most highly induced *HiGH61* (*HiGH61A*, *HiGH61B*, *HiGH61D* and *HiGH61G–HiGH61I*) genes showed the highest observed transcript levels on spruce natural substrates, higher on spruce hearth wood than in spruce reaction zone wood possibly reflecting is pivotal role in the decomposition of cellulose during this preferred saproptrophic mode in this host. *HiGH61A*, *HiGH61B*, *HiGH61D*, and *HiGH61G–HiGH61I* are induced on pine sapwood, while close to constitutive levels is detected on pine heartwood (resinous wood with antifungal properties), possibly reflecting that colonization of the pine sapwood is the predominant growth mode in this host. The additional two *GH61* genes (*HIGH61C* and *HIGH61J*) appeared to be coordinately expressed with *HiLcc2* (encoding a laccase), leading us to speculate that these GH61s may play a role beyond cleaving cellulose. The common upregulated transcription patterns of most *GH61* genes together with several of the reference cellulolytic and hemicellulolytic *GH*s (GH5 and GH7 cellulases) during growth on the natural woody substrates reflect the co-metabolic nature of cell wall polymer degradation.

Sequence and phylogenetic analysis of the *H. irregulare* GH61s shows high sequence divergence (Figs. 1 and 3). Similar divergence has been shown for other ascomycetes and basidiomycetes fungi (Harris et al. 2010), indicating that much of the duplication and divergence of the genes encoding GH61 proteins is a fairly ancient evolutionary event. This diversity has been largely maintained and in some cases apparently amplified during this evolutionary span, suggesting a significant selective pressure to retain a heterogeneous collection of *GH61*-encoding genes in cellulose-degrading fungi (Harris et al. 2010). The high level of indel and single-nucleotide polymorphism observed when comparing *GH61*s between the closely related *H. irregulare* and *H. parviporum* species (Table S6 in the ESM) is compatible with this hypothesis. Generally white-rot fungi have more of GH61 family proteins than brown-rot fungi, and this also applies for *H. irregulare* which possessed ten members in the gene family, whereas the reference brown rot fungi *P. placenta* and *S. lacrymans* have four and five members, respectively (Table S3 in the ESM). Among white-rot fungi, the necrotrophic and saprotrophic *H. irregulare* possesses the lower number of GH61 family genes (10) in comparison with saprotrophic *P. chrysosporium* (14), *P. ostreatus* (29), and *S. commune* (22). Notably, fungi growing on hardwoods (non-monocot angiosperm trees) generally possess more GH61 family genes than fungal species growing mostly on softwoods (wood from conifers that are gymnosperms), like *H. irregulare* and *P. carnosa* (this could be due to the more complex cell wall chemistry and that the lignin composition and their links to cellulose is more complex in angiosperm trees).

Detailed analysis of the *H. irregulare* GH61 polypeptides enabled identification of a region in the GH61 sequences that may contain insertions/deletions (Figs. 1 (yellow-colored part of MSA) and 2a). This region provides an extension of the protein surface next to the metal-binding site (Fig. 2; also observed for HjGH61B), putatively providing means of substrate binding as this region also contains exposed aromatic amino acids that are known to interact with carbohydrates. A second insertion is observed in the HiGH61H, HiGH61G, HiGH61I, and HiGH61E sequences close to the second conserved His in the metal-binding motif (Fig. 1 (blue-colored part of MSA)), which may also expand the putative substrate-binding surface residues important for GH61 activity clustered at the putative reactive surface of the GH61s (Fig. 2b; His20, His92, His167, Gln176, Tyr178, and Tyr217—HiGH61A numbering) are conserved in all proteins except HiGH61G and HiGH61J (Fig. 2). Interestingly, HiGH61G has most of these residues substituted with amino acids that are likely to be incompatible with the cellulose oxidizing activity proposed for proteins in the GH61 family (His20→Arg, His92→Glu, His167→Gln, and Tyr178→Phe, HiGH61A numbering; Figs. 1 and 2b; (Vaaje-Kolstad et al. 2010) while HiGH61J shows two substitutions of these conserved residues; Gln176→Glu and Tyr217→Phe.

It is intriguing to note that *HiGH61G* is highly upregulated on several substrates (Fig. 4) since this protein lacks amino acids essential for the predicted GH61 oxidative activity (the sequence of this gene was verified by sequencing of PCR products from two species, independent isolates). It may be that this protein has evolved a different enzymatic function or even a non-enzymatic role compared with other GH61 enzymes. The putative enzymatic properties of *Hi*GH61G should be further characterized in order to reveal the functional significance of this protein.

Recent work (Langston et al. 2011) demonstrated that cellobiose dehydrogenase can provide electrons to GH61s to form an oxidoreductive system for microbial lignocellulose degradation. The two *CDH* genes of *H. irregulare* differ significantly by domain structure. *HiCDH1* showed substrate-specific transcript level profiles and appeared to be coordinately regulated with different subgroups of *HiGH61* genes, whereas *HiCDH2* appeared not to be significantly transcribed on any substrates (Fig. 5) Interestingly, similar results have been observed for the filamentous ascomycete *Neurospora crassa* which also have two genes encoding *CDHs*, whereof only one is expressed (Phillips et al. 2011). The expressed *N. crassa CDH* (*CDH-1*; Uniprot ID Q7RXM0), which has been suggested to be the designated electron donor for the *N. crassa* GH61s, also shares the domain structure of the *H. irregulare* protein HiCDH1 (these genes share ∼60 % sequence identity). It is possible that HiCDH1 of *H. irregulare* can play a similar role to that of CDH1 from *N. crassa* in biomass degradation. The second CDH from H. irregulare (HiCDH2) also has a similar domain structure to the second CDH from *N. crassa*, which only contains one flavin domain and a C-terminal CBM1. Interestingly, neither the *HiCDH2* nor the *N. crassa CDH2* are expressed during fungal growth on cellulose (Fig. 5) (Phillips et al. 2011).

The generally higher transcript levels of *GH61* genes on wood substrates in comparison to artificial media (MacDonald et al. 2011) suggest involvement of these genes in degradation of wood fiber lignocelluloses. Differential regulation of *GH61* family genes on different substrates also imply specificity of different *GH61* polysaccharide oxidases to different types of wood cell-wall compounds and could determine the hosts species specificity of a particular fungi species. The fact that some of the *GH61* genes, such as *HiGH61A* and *HiGH61B*, showed marked difference in the transcript level between spruce heartwood and pine sapwood may reflect some tree-specific differences in the structure or composition of the tracheid cell wall. For natural substrates, the currently employed experimental system should provide artificially accelerated decay conditions due to the involved pulverizing of wood and use of inoculum high in colony forming units. The generally lower transcript levels of *HiGH61* genes on spruce reaction zone and pine heartwood compared with spruce heartwood and pine sapwood, respectively, are presumably related to the high concentration of antifungal compounds in spruce reaction zone and pine heartwood (Woodward et al. 1998). For example, in the reaction zone of Norway spruce the fungus is likely to use its ligninolytic repertoire for detoxification of host defense polyphenols as well, this resource allocation affecting negatively the rate of cell wall degradation and exposure of cellulose to enzymatic activity (Yamada 2001).

In summary, there is high diversity among the ten *GH61* genes present in *H. irregulare*. Based on the transcript profile patterns on the included natural and defined substrates, these genes could be divided into two groups. The major group consisted of eight genes that showed coordinated gene expression with the included reference cellulolytic and hemicellulolytic GHs and generally high up-regulation level on woody substrates in contrast to the defined cellulose medium. This demonstrates the co-metabolic nature of lignocellulose degradation. The remaining two GH61s, together with starch and oligosaccharide active reference genes, showed little regulation in comparison with the basal Hagem medium control, and could be involved in degradation of more easily available non-structural carbohydrates that are particularly important during the initial exploratory colonization phase of host tissue by a microbe. These data suggest that GH61 genes participate in degradation of both the structural and reserve carbohydrates of xylem. Further research on both *GH61* genes and proteins are warranted particularly on the highly expressed *HiGH61G* with a widely divergent catalytic site and whether specific GH61s may have a role in breaking the link between cellulose and lignin.

## Electronic supplementary material

Below is the link to the electronic supplementary material.

ESM 1 (DOC 399 kb)
