# Supplementary material for: Substrate-specific transcription of the enigmatic GH61 family of the pathogenic white-rot fungus Heterobasidion irregulare during growth on lignocellulose
Source: Appl Microbiol Biotechnol. 2012 Jun 21;95(4):979–90. doi: 10.1007/s00253-012-4206-x (PMC3405238; doi:10.1007/s00253-012-4206-x)
Supplement: Supplementary file 1 — (DOC 399 kb) [file 253_2012_4206_MOESM1_ESM.doc]

# Supplementary material

# Substrate specific transcription of the enigmatic GH61 family of the pathogenic white-rot fungus *Heterobasidion* *irregulare* during growth on lignocellulose

Igor Yakovlev^1^, Gustav Vaaje-Kolstad^2^, Ari M. Hietala^1^, Emil Stefańczyk^1^, Halvor Solheim^1^, Carl Gunnar Fossdal^1†^

^1^Norwegian Forest and Landscape Institute, P.O. Box 115, N-1431 Ås, Norway; ^2^Department of Chemistry, Biotechnology and Food Science, Norwegian University of Life Sciences, N-1432, Ås, Norway

^†^Corresponding author: Carl Gunnar Fossdal

e-mail: foc@skogoglandskap.no

Table S1. General description of the glycosyl hydrolase family 61 (*GH61*) genes and proteins in the *H. irregulare* genome*

| Gene ID** | Genome location | Gene model | Protein ID  (JGI) | Signal peptide length | Strand | Number of exons | gDNA length (bp) | CDS length (bp) | Protein length (aa) |
| --- | --- | --- | --- | --- | --- | --- | --- | --- | --- |
| *Hi****GH61A*** | [scaffold_10:1211236-1212334](http://genome.jgi-psf.org/cgi-bin/browserLoad?db=Hetan2&position=scaffold_10:1211236-1212334) | Genemark.6536_g | 105463 | 19 | + | 8 | 1099 | 699 | 233 |
| *Hi****GH61B*** | [scaffold_01:1724543-1725558](http://genome.jgi-psf.org/cgi-bin/browserLoad?db=Hetan2&position=scaffold_01:1724543-1725558) | EuGene1000694 | 166613 | 19 | + | 6 | 1016 | 735 | 245 |
| *Hi****GH61C*** | [scaffold_10:1078504-1079597](http://genome.jgi-psf.org/cgi-bin/browserLoad?db=Hetan2&position=scaffold_10:1078504-1079597) | dfl_YAI_gw1.8.895.1 | 181229 | 19 | + | 9 | 1094 | 702 | 234 |
| *Hi****GH61D*** | [scaffold_13:1268149-1269890](http://genome.jgi-psf.org/cgi-bin/browserLoad?db=Hetan2&position=scaffold_13:1268149-1269890) | estExt_Genewise1Plus.C_130017 | 67582 | 20 | – | 9 | 1742 | 966 | 322 |
| *Hi****GH61E*** | [scaffold_03:3214244-3215278](http://genome.jgi-psf.org/cgi-bin/browserLoad?db=Hetan2&position=scaffold_03:3214244-3215278) | [e_gw1.18.64.1](http://shake.jgi-psf.org/cgi-bin/dispGeneModel?db=Hetan1&tid=58346) | 58346 | 16 | – | 6 | 1035 | 747 | 249 |
| *Hi****GH61F*** | [scaffold_08:582672-583874](http://genome.jgi-psf.org/cgi-bin/browserLoad?db=Hetan2&position=scaffold_08:582672-583874) | Genemark.5169_g | 104096 | 21 | – | 9 | 1203 | 759 | 253 |
| *Hi****GH61G*** | [scaffold_05:1168808-1169991](http://genome.jgi-psf.org/cgi-bin/browserLoad?db=Hetan2&position=scaffold_05:1168808-1169991) | EuGene7000434 | 173373 | 20 | + | 9 | 1184 | 723 | 241 |
| *Hi****GH61H*** | [scaffold_07:1260325-1262035](http://genome.jgi-psf.org/cgi-bin/browserLoad?db=Hetan2&position=scaffold_07:1260325-1262035) | estExt_Genewise1Plus.C_50409 | 63659 | 17 | – | 11 | 1711 | 1029 | 343 |
| *Hi****GH61I*** | [scaffold_05:1166617-1168178](http://genome.jgi-psf.org/cgi-bin/browserLoad?db=Hetan2&position=scaffold_05:1166617-1168178) | [estExt_Genewise1Plus.C_70589](http://genome.jgi-psf.org/cgi-bin/dispGeneModel?db=Hetan2&tid=64994) | 64994 | 19 | + | 10 | 1562 | 948 | 316 |
| *Hi****GH61J*** | [scaffold_09:2096595-2098288](http://genome.jgi-psf.org/cgi-bin/browserLoad?db=Hetan2&position=scaffold_09:2096595-2098288) | estExt_Genewise1Plus.C_160208 | 68440 | 22 | – | 4 | 1694 | 999 | 333 |

* – http://genome.jgi-psf.org/Hetan2/Hetan2.home.html *H. irregulare* genome v.2; **– *H. irregulare* (*Hi*) *GH61* polysaccharide oxidase gene name

Table S2. Matrix of similarity and identity (%) between protein sequences of GH61 polysaccharide oxidases from *Heterobasidion* *irregulare* (Similarity (lower triangle)/Identity (upper triangle))

|  | Hi**GH61A** | Hi**GH61B** | Hi**GH61C** | Hi**GH61D** | Hi**GH61E** | Hi**GH61F** | Hi**GH61G** | Hi**GH61H** | Hi**GH61I** | Hi**GH61J** |
| --- | --- | --- | --- | --- | --- | --- | --- | --- | --- | --- |
| 1. Hi**GH61A** | x | 30.9 | **78.6** | 23.6 | 36.3 | 37.8 | 39.1 | 27.0 | 28.6 | 26.3 |
| 2. Hi**GH61B** | 48.0 | x | 33.1 | 41.2 | 34.3 | 37.7 | 33.8 | 25.4 | 29.5 | 24.1 |
| 3. Hi**GH61C** | **86.3** | 50.4 | x | 25.5 | 34.0 | 36.5 | 34.6 | 27.3 | 29.0 | 24.6 |
| 4. Hi**GH61D** | 35.5 | 57.6 | 36.4 | x | 28.1 | 29.5 | 26.9 | 38.4 | 39.2 | 32.3 |
| 5. Hi**GH61E** | 49.6 | 52.8 | 48.0 | 41.4 | x | 38.1 | 55.6 | 34.0 | **61.1** | 24.8 |
| 6. Hi**GH61F** | 49.6 | 55.6 | 50.0 | 42.4 | 57.1 | x | 34.5 | 25.9 | 31.3 | 27.4 |
| 7. Hi**GH61G** | 55.4 | 54.1 | 54.2 | 38.6 | 69.4 | 50.8 | x | 31.7 | 46.2 | 22.7 |
| 8. Hi**GH61H** | 37.7 | 37.4 | 38.9 | 53.5 | 45.3 | 39.5 | 42.1 | x | 45.7 | 30.2 |
| 9. Hi**GH61I** | 40.6 | 45.1 | 40.6 | 57.6 | 67.3 | 44.8 | 56.2 | 59.6 | x | 30.2 |
| 10. Hi**GH61J** | 39.2 | 40.7 | 37.3 | 50.9 | 40.1 | 41.3 | 37.0 | 48.0 | 49.7 | x |

Table S3. Comparison of numbers of glycosyl hydrolase family 61 genes in the genomes of several wood degrading basidiomycetes fungi (based on cluster analyses in JGI genome browser*)

| Gene families | *Heterobasidion irregulare* | *Phanerochaete carnosa* | *Phanerochaete chrysosporium* | *Pleurotus ostreatus* | *Schizophyllum commune* | *Postia placenta* | *Serpula lacrymans* | *Coprinopsis cinerea* | *Laccaria bicolor* |
| --- | --- | --- | --- | --- | --- | --- | --- | --- | --- |
|  | White–rot necrotroph | White–rot saprotroph | White–rot saprotroph | White–rot saprotroph | White–rot saprotroph | Brown–rot saprotroph | Brown–rot saprotroph | Soil and leaf-litter saprotroph | Ectomycorrhi-zal, symbiotic |
| Genome size, Mbp | 33.6 | 46.29 | 35.1 | 34.3 | 38.5 | 90.9 | 42.7 | 37.5 | 64.9 |
| Number of genes | 13 405 | 13 937 | 10 048 | 11 603 | 13 210 | 9 113 | 12 789 | 13 544 | 19 036 |
| GH61 genes | 10 | 11 | 14 | 29 | 22 | 4 | 5 | 35 | 13 |

* – http://genome.jgi-psf.org/clustering/pages/cluster/clusters.jsf?organism=Hetan2

Table S4 Amplicon size and primers used for transcript level profiling of GH family 61 genes and selected reference genes of *H. irregulare* (*Hi*) involved in lignocellulose degradation

| Gene ID | Protein ID(JGI)^*^ | Forward primer (5′→ 3′)^**^ | Reverse primer (5′→ 3′)^**^ | Amplicon size, bp |
| --- | --- | --- | --- | --- |
| *HiGH61A* | 105463 | CCCGTCGTCGCCATTCCTG | GGCCCGGCTGCGTGTAGTTG | 97 |
| *HiGH61B* | 166613 | GGTTGCCGCACATGGTGGTGT | TCGCGCTGGATGGTGCTTTG | 105 |
| *HiGH61C* | 181229 | TCACCGTCGTCGCTGGCACA | CCGAACCGTCGAACGTGGAGA | 120 |
| *HiGH61D* | 67582 | GCTGCCTCGCCGACCTCATC | CCATCCGATCCCGCCACACT | 90 |
| *HiGH61E* | 58346 | CCAACAAGGGCAGGGTCACG | GCAGCATGCAGGGCGATAAT | 94 |
| *HiGH61F* | 104096 | TTGCGGAGGCTGGGAAGACG | GCCTGGAGGTTGGCAGGAAC | 103 |
| *HiGH61G* | 173373 | GTGACCGCCTCATTCCACAAGAC | TCAGGGACGACGGCTAGGTACGC | 113 |
| *HiGH61H* | 63659 | CGCCCTCCTCGTCGGCTAGT | GATCCCGCCGCACTGACCAT | 119 |
| *HiGH61I* | 64994 | TCGTCGTATCCAGGTGCCCAGTT | TGGGTCGGTTCCGCTGTATGC | 117 |
| *HiGH61J* | 68440 | GCCCGGCCATGTCGAATCTG | GGCTGTCCTTTGGCGCTGGT | 114 |
| *HiGH3.1* | 124437 | TCGACAGCAGCCATGACCAAA | GTTGTACGCAGGGCGGTGGA | 116 |
| *HiGH3.3* | 107773 | TGGATGTGAAGAACACGGGCAAG | CCTTCAACGGAGCAGGCGATG | 93 |
| *HiGH5.1* | 66839 | CTCGTGGCTCCAGGCTAACAACC | AGACGCCGCTCTGCTGCATCG | 117 |
| *HiGH5.2* | 62063 | CGACGATCTCTGCGTACATCAAATCC | CGATACCCTCGCCTCCTTGGT | 120 |
| *HiGH6.1* | 60114 | TCACACAGGGCAACCCGAACTATG | CGACAATGAACTGGGCTGGGAAG | 96 |
| *HiGH7* | 38802 | CAAGGGCGTCGTGCTGGTGA | TGGAGGCGGACGAGGTGGTG | 95 |
| *HiGH15* | 36572 | CGGTTCCGTCGATGCTCTGC | ACGAGCTGGCCGGGAGATTG | 107 |
| *HiGH27* | 101995 | CTTCGTCCACGTCCGCGACC | GTGAATGTTGTGAGGCTGGCTCC | 109 |
| *HiGH88.1* | 171916 | CCATGCGAACAACGCGACGA | TGGACGTGTGCCCTCCCAAAG | 101 |
| *HiCDH1* | 157537 | GGGATGCTCATGTCGGCGGTAG | CCTTGCCTTCTGCCTGGGTTC | 103 |
| *HiCDH2* | 122297 | GCGGGCTATGTCCAACCTTTGCT | CCGGAACGACGCCTTGATATGTGT | 96 |
| *HiHFB1* | 17575 | GACGGCTTCCGCCACCTCTG | TGAGCCAAACCCTCCCGTGT | 118 |
| *HiHFB2* | 65822 | TCTCCTCGGCATCGTCCTGAA | GACGGTTTGTGATACGCAGTTGGA | 109 |
| *HiCEP1* | 106207 | CGAACCGCAAGGGAGGGTGA | GGCATTGCTCATTCAATTTCGTTCA | 101 |
| *HiAao2* | 163945 | CGATTCGAAGGAGGGCGTGCT | TGCGCAGCCGTCAAGAAAGG | 102 |
| *HiQOr4* | 63899 | GACCAGCCTCGACCCGTTGG | CGGCATCGCCGGGATATTGA | 100 |
| *HiαTub1* | 62388 | ACGTCGGTGAGGGCATGGAG | GCAGAGTCGATGCCGACCTCCT | 97 |
| *HiAct1* | 147003/ 408649 | GCGCCTCCCGAGCGGAAGTA | CCAGGGCCCGACTCGTCGTA | 113 |
| *HiUbc2* | 145590 | ACGAGTGGTCGCCCGTCCTG | AGCGGTCGTTGCCCTCTGGT | 102 |

^*^ – http://genome.jgi-psf.org/Hetan2/Hetan2.home.html;^**^ – All primers (listed in 5′ to 3′ orientation) were designed with Primer3 software (http://frodo.wi.mit.edu/cgi-bin/primer3/primer3_www.cgi#disclaimer) (Rozen and Skaletsky 2000) and synthesized by Invitrogen; *Lcc* – laccase; *Aao* – aryl-alcohol oxidase; *QOr* – Quinone oxidoreductase; *СDH* – cellobiose dehydrogenase; *HBF* – hydrophobin; *GH* -– glycoside hydrolase


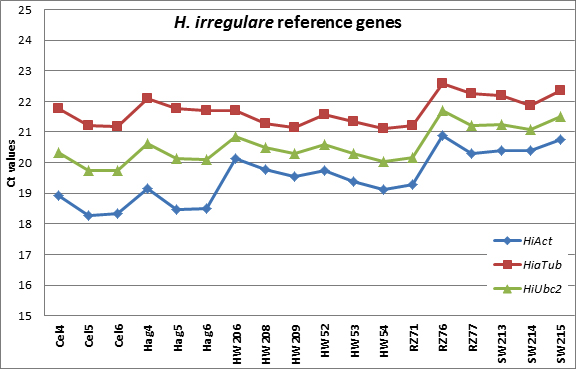


Fig. S1. *H. irregulare* endogenous reference gene transcripts used in qRT-PCR. Expression patterns of selected *H. irregulare* reference genes [actin (*HiAct*), α-tubulin (*HiαTub*) and ubiquitin-conjugating enzyme 2 (*HiUbc2*)] used for qRT-PCR analysis. Ct values are shown as average between three sample replicates per plate for each treatment. The substrates used were cDNA generated from total RNA from all experimental conditions examined in this study. Cellulose (Cel4-6), Hagem medium alone (Hag4-6), Heartwood from spruce (HW206-209), Heartwood from pine (HW52-53), Reaction zone wood from spruce (RZ71, 76 and 77)) and sap wood from pine (SW213-215).

Table S5. Description of primers used for cloning of GH family 61 genes of *H. irregulare* and *H. parviporum*

| Gene ID | Forward primers (5′→ 3′)^*^ | Reverse primers (5′→ 3′)^*^ | Fragment size, bp | Comments |
| --- | --- | --- | --- | --- |
| *GH61A* | ATCGGATGTTTGAAGGATGG | GCTGACTTGCGGCTCATACA | 794 |  |
| *GH61B* | GTTCCTAGTCCCCCTTCTGG | GGTTCCGCTTCATTATCTGG | 867 |  |
| *GH61C* | GACATTACTGCTCGGTCCTG | GGTTCCGCTTCATTATCTGG | 814 | Not cloned |
| *GH61D* | TCGCACCCAACCTCGCTCAA | TGGACGACAAGGGCCACAATG | 1103 |  |
| *GH61E* | CCTGGTCAGTGTTGGGATTC | CGCAACCATACTTTCAGCAA | 848 | Not cloned |
| *GH61F* | CAGGCGTACAGAACAGCGTA | CACTCGCCCTCTTTCTCAAC | 796 |  |
| *GH61G* | TCAAACGTGTTCTGTGACGA | CCGATGATCAGCAAGTGAAA | 827 |  |
| *GH61H* | ACACACCTTTGCTCGGATTT | GGAGGCATTTCATTGAGCAT | 1139 |  |
| *GH61I* | TATAACCTGGCGAGGAATGG | TGACCACTGGTAGGAGGATG | 1068 |  |
| *GH61J* | GCTCCCTCCATACATTCCTCA | CAGTGTCGTGTCCCAGCGTA | 1114 |  |

^*^ – All primers (listed in 5′ to 3′ orientation) were designed with Primer3 software (http://frodo.wi.mit.edu/cgi-bin/primer3/primer3_www.cgi#disclaimer) (Rozen and Skaletsky 2000) and synthesized by Invitrogen.

Table S6. Summary of the coding sequence comparison of GH, family 61 genes between *H. irregulare* and *H. parviporum*

| Gene ID | Database ID | | Cloning success | | Remarks | |
| --- | --- | --- | --- | --- | --- | --- |
|  | *H. parviporum^*^* | *H. irregulare^**^* | *H. parviporum* | *H. irregulare* | *H. parviporum* | *H. irregulare* |
| *GH61A* | JQ290102 | 105463 | + Two bands | + | One fragment matches to GH61A, 29 NS*^**^*^*^ in CDS (incl. 5 non synonymous) | Confirmed |
| *GH61B* | JQ290103 | 166613 | +  Two bands | + | One fragment matches to GH61B, 49 NS in CDS (incl. 10 non synonymous) | Confirmed |
| *GH61C* | – | 181229 | – | – | No amplification | No amplification |
| *GH61D* | JQ290104 | 67582 | + | + | 46 NS in CDS (incl. 6 non synonymous) | Confirmed |
| *GH61E* | – | 58346 | – | – | No amplification | No amplification |
| *GH61F* | JQ290105 | 104096 | +  Two bands | +  Two bands | One fragment matches to GH61F, 32 NS in CDS (incl. 10 non synonymous) | Confirmed. In one sequence variant there is no excised intron 3 |
| *GH61G* | JQ290106 | 173373 | + | + | 23 NS in CDS (incl. 8 non synonymous) | Confirmed |
| *GH61H* | JQ290107 | 63659 | + | + | 53 NSs in CDS (incl. 15 non synonymous) | Confirmed |
| *GH61I* | JQ290108 | 64994 | + | + | 36 NSs in CDS (incl. 4 non synonymous). In one sequence variant there is 21 bp synonymous insert (TTCTCCTACGAGCTCTTCTGC – SPTSSSA) | Confirmed |
| *GH61J* | JQ290109 | 68440 | + | + | 48 NSs in CDS (incl. 10 non synonymous) and indels in 5ʹ UTR | Confirmed |

^*^ – GenBank accession number;^**^ – http://genome.jgi-psf.org/Hetan2/Hetan2.home.html;*^***^ –* NS – nucleotide substitution

Table S7. Correlation coefficients^*^ between the qRT-PCR transcriptional changes of the *GH61s* and selected cellulose active *H. irregulare* genes during growth on the different substrates.

|  | ***GH61A*** | ***GH61B*** | ***GH61C*** | ***GH61D*** | ***GH61E*** | ***GH61F*** | ***GH61G*** | ***GH61H*** | ***GH61I*** | ***GH61J*** | ***Lcc2*** | ***Aao2*** | ***GH88.1*** | ***QOR4*** | ***GH5.1*** | ***GH5.2*** | ***GH7*** | ***GH15*** | ***GH27*** | ***HBF1*** | ***HBF2*** | ***CDH1*** | ***CDH2*** | ***GH3.1*** |
| --- | --- | --- | --- | --- | --- | --- | --- | --- | --- | --- | --- | --- | --- | --- | --- | --- | --- | --- | --- | --- | --- | --- | --- | --- |
| ***GH61A*** | **1** |  |  |  |  |  |  |  |  |  |  |  |  |  |  |  |  |  |  |  |  |  |  |  |
| ***GH61B*** | 0.957 | **1** |  |  |  |  |  |  |  |  |  |  |  |  |  |  |  |  |  |  |  |  |  |  |
| ***GH61C*** | -0.562 | -0.472 | **1** |  |  |  |  |  |  |  |  |  |  |  |  |  |  |  |  |  |  |  |  |  |
| ***GH61D*** | 0.827 | 0.895 | -0.344 | **1** |  |  |  |  |  |  |  |  |  |  |  |  |  |  |  |  |  |  |  |  |
| ***GH61E*** | 0.931 | 0.984 | -0.402 | 0.844 | **1** |  |  |  |  |  |  |  |  |  |  |  |  |  |  |  |  |  |  |  |
| ***GH61F*** | 0.763 | 0.729 | -0.011 | 0.633 | 0.700 | **1** |  |  |  |  |  |  |  |  |  |  |  |  |  |  |  |  |  |  |
| ***GH61G*** | 0.897 | 0.982 | -0.422 | 0.942 | 0.962 | 0.659 | **1** |  |  |  |  |  |  |  |  |  |  |  |  |  |  |  |  |  |
| ***GH61H*** | 0.983 | 0.963 | -0.534 | 0.912 | 0.924 | 0.737 | 0.9331 | **1** |  |  |  |  |  |  |  |  |  |  |  |  |  |  |  |  |
| ***GH61I*** | 0.924 | 0.904 | -0.514 | 0.950 | 0.845 | 0.670 | 0.9013 | 0.9766 | **1** |  |  |  |  |  |  |  |  |  |  |  |  |  |  |  |
| ***GH61J*** | -0.251 | -0.220 | 0.037 | 0.126 | -0.218 | -0.540 | -0.095 | -0.113 | 0.0607 | **1** |  |  |  |  |  |  |  |  |  |  |  |  |  |  |
| ***Lcc2*** | -0.558 | -0.483 | 0.691 | -0.326 | -0.363 | -0.463 | -0.405 | -0.508 | -0.452 | 0.590 | **1** |  |  |  |  |  |  |  |  |  |  |  |  |  |
| ***Aao2*** | -0.445 | -0.370 | 0.490 | -0.389 | -0.211 | -0.496 | -0.331 | -0.454 | -0.475 | 0.404 | 0.907 | **1** |  |  |  |  |  |  |  |  |  |  |  |  |
| ***GH88.1*** | -0.467 | -0.415 | 0.332 | -0.194 | -0.330 | -0.646 | -0.316 | -0.385 | -0.280 | 0.861 | 0.892 | 0.807 | **1** |  |  |  |  |  |  |  |  |  |  |  |
| ***QOR4*** | -0.394 | -0.317 | 0.562 | -0.424 | -0.146 | -0.337 | -0.308 | -0.438 | -0.507 | 0.152 | 0.828 | 0.963 | 0.626 | **1** |  |  |  |  |  |  |  |  |  |  |
| ***GH5.1*** | 0.874 | 0.909 | -0.275 | 0.971 | 0.856 | 0.797 | 0.923 | 0.935 | 0.950 | -0.051 | -0.398 | -0.468 | -0.344 | -0.452 | **1** |  |  |  |  |  |  |  |  |  |
| ***GH5.2*** | 0.634 | 0.684 | 0.102 | 0.818 | 0.622 | 0.851 | 0.711 | 0.708 | 0.742 | -0.129 | -0.278 | -0.476 | -0.373 | -0.421 | 0.900 | **1** |  |  |  |  |  |  |  |  |
| ***GH7*** | 0.618 | 0.669 | -0.001 | 0.888 | 0.594 | 0.704 | 0.727 | 0.728 | 0.810 | 0.129 | -0.217 | -0.450 | -0.197 | -0.471 | 0.915 | 0.960 | **1** |  |  |  |  |  |  |  |
| ***GH15*** | -0.069 | -0.158 | -0.347 | 0.175 | -0.260 | -0.359 | -0.091 | 0.050 | 0.240 | 0.760 | 0.009 | -0.219 | 0.380 | -0.464 | 0.058 | -0.032 | 0.220 | **1** |  |  |  |  |  |  |
| ***GH27*** | -0.096 | -0.006 | 0.825 | 0.068 | 0.016 | 0.521 | 0.015 | -0.075 | -0.088 | -0.304 | 0.245 | 0.041 | -0.153 | 0.190 | 0.206 | 0.576 | 0.407 | -0.489 | **1** |  |  |  |  |  |
| ***HBF1*** | -0.127 | -0.366 | -0.259 | -0.231 | -0.410 | -0.309 | -0.415 | -0.115 | -0.008 | 0.467 | 0.074 | -0.042 | 0.311 | -0.198 | -0.239 | -0.309 | -0.163 | 0.741 | -0.484 | **1** |  |  |  |  |
| ***HBF2*** | 0.079 | -0.195 | -0.492 | -0.202 | -0.262 | -0.107 | -0.300 | 0.038 | 0.084 | 0.099 | -0.308 | -0.326 | -0.087 | -0.409 | -0.158 | -0.273 | -0.195 | 0.567 | -0.549 | 0.907 | **1** |  |  |  |
| ***CDH1*** | 0.716 | 0.816 | -0.668 | 0.754 | 0.743 | 0.398 | 0.843 | 0.744 | 0.715 | -0.195 | -0.689 | -0.573 | -0.502 | -0.568 | 0.696 | 0.455 | 0.489 | 0.004 | -0.254 | -0.429 | -0.216 | 1 |  |  |
| ***CDH2*** | -0.452 | -0.385 | 0.308 | -0.261 | -0.270 | -0.664 | -0.303 | -0.403 | -0.345 | 0.741 | 0.895 | 0.907 | 0.971 | 0.761 | -0.410 | -0.479 | -0.336 | 0.194 | -0.191 | 0.192 | -0.167 | -0.237 | **1** |  |
| ***GH3.1*** | 0.172 | 0.065 | 0.067 | 0.081 | 0.168 | -0.040 | 0.034 | 0.169 | 0.187 | 0.491 | 0.576 | 0.608 | 0.633 | 0.525 | 0.059 | -0.084 | 0.002 | 0.184 | -0.152 | 0.478 | 0.266 | -0.630 | 0.639 | **1** |

^*^ – Red figures on red background - correlation coefficients >0.75; Brown figures on yellow background - correlation coefficients >0.6; Pink figures on pink background - correlation coefficients <-0.6

Table S8. p-values for transcriptional changes for *H. irregulare* transcripts*

|  | **Transcript** | **p-value (comparing to control - Hagem media)** | | | | |
| --- | --- | --- | --- | --- | --- | --- |
|  |  | **Ps_HW** | **Pa_HW** | **Pa_RZ** | **Ps_SW** | **Pa_Cel** |
| 1 | *HiGH61A* | 0.898087 | 0.000102 | 0.002076 | 0.000707 | 0.000034 |
| 2 | *HiGH61B* | 0.024974 | 0.000482 | 0.013268 | 0.000722 | 0.000547 |
| 3 | *HiGH61C* | 0.000155 | 0.000156 | 0.000157 | 0.382352 | 0.025037 |
| 4 | *HiGH61D* | 0.200253 | 0.001505 | 0.000115 | 0.001625 | 0.000334 |
| 5 | *HiGH61E* | 0.000034 | 0.022516 | 0.311799 | 0.000036 | 0.000081 |
| 6 | *HiGH61F* | 0.000008 | 0.000381 | 0.022559 | 0.706268 | 0.000046 |
| 7 | *HiGH61G* | 0.000564 | 0.000307 | 0.012268 | 0.000933 | 0.000711 |
| 8 | *HiGH61H* | 0.028983 | 0.000177 | 0.010099 | 0.001011 | 0.006572 |
| 9 | *HiGH61I* | 0.000366 | 0.000755 | 0.003956 | 0.002045 | 0.000137 |
| 10 | *HiGH61J* | 0.00009 | 0.000424 | 0.002824 | 0.126608 | 0.000135 |
| 11 | *HiLcc2* | 0.000241 | 0.001471 | 0.000893 | 0.194076 | 0.012792 |
| 12 | *HiAao2* | 0.000037 | 0.000038 | 0.000037 | 0.001874 | 0.000151 |
| 13 | *HiGH88.1* | 0.000025 | 0.000067 | 0.000056 | 0.816273 | 0.000203 |
| 14 | *HiQOR4* | 0.00003 | 0.000062 | 0.000032 | 0.00004 | 0.000104 |
| 15 | *HiGH5.1* | 0.118604 | 0.005017 | 0.000028 | 0.0049 | 0.000016 |
| 16 | *HiGH5.2* | 0.003208 | 0.001441 | 0.00471 | 0.004299 | 0.000108 |
| 17 | *HiGH7* | 0.000065 | 0.001039 | 0.000013 | 0.002561 | 0.004412 |
| 18 | *HiGH15* | 0.000791 | 0.041389 | 0.000843 | 0.000377 | 0.001426 |
| 19 | *HiGH27* | 0.00036 | 0.112187 | 0.003019 | 0.004964 | 0.017416 |
| 20 | *HiHBF1* | 0.705832 | 0.000291 | 0.161972 | 0.017413 | 0.003618 |
| 21 | *HiHBF2* | 0.058957 | 0.001576 | 0.079656 | 0.010587 | 0.001466 |
| 22 | *HiCDH1* | 0.000105 | 0.005997 | 0.000284 | 0.000061 | 0.00082 |
| 23 | *HiCDH2* | 0.000288 | 0.000288 | 0.000251 | 0.241547 | 0.000499 |
| 24 | *HiGH3.1* | 0.00002 | 0.012311 | 0.882006 | 0.150161 | 0.00003 |

*–The qRT-PCR data were analyzed using the RT^2^ Profiler PCR Array Data Analysis Version 3.5 from SABiosiences/Qiagen (Frederick, MD, USA) using the default settings. RT^2^ Profiler PCR Array Data Analysis Version 3.5p calculated the p-values based on Student’s t-test of the replicate 2^( - Delta Ct) values for each gene in the control group and treatment groups. p values less than 0.05 are indicated in red. Only the transcriptional changes greater than 2-fold increase or decrease and p-value less than 0.05 should be considered significant.

### References

Rozen S, Skaletsky HJ (2000) Primer3 on the WWW for general users and for biologist programmers. In: Krawetz S, Misener S (eds) Methods in Molecular Biology. Humana Press, Totowa, NJ, pp 365-386
